# (2,2-Bipyrid­yl)bis­(η^5^-penta­methyl­cyclo­penta­dien­yl)strontium(II)

**DOI:** 10.1107/S1600536808024677

**Published:** 2008-08-06

**Authors:** Daniel Kazhdan, Yung-Jin Hu, Akos Kokai, Zerubba Levi, Sergio Rozenel

**Affiliations:** a Chemistry Department and Chemical Sciences Division of Lawrence Berkeley National Laboratory, University of California, Berkeley, California 94720, USA; b College of Chemistry, University of California at Berkeley, Berkeley, California 94720, USA

## Abstract

In the title compound, [Sr(C_10_H_15_)_2_(C_10_H_8_N_2_)], the Sr—N distances are 2.624 (3) and 2.676 (3) Å, the Sr⋯Cp ring centroid distances are 2.571 and 2.561 Å and the N—C—C—N torsion angle in the bipyridine ligand is −2.2 (4)°. Inter­estingly, the bipyridine ligand is tilted. The angle between the plane defined by the Sr atom and the two bipyridyl N atoms and the plane defined by the 12 atoms of the bipyridine ligand is 10.7 (1)°.

## Related literature

For related literature, see: Allen (2002 ▶); Burns & Andersen (1987 ▶); Schultz *et al.* (2002 ▶).
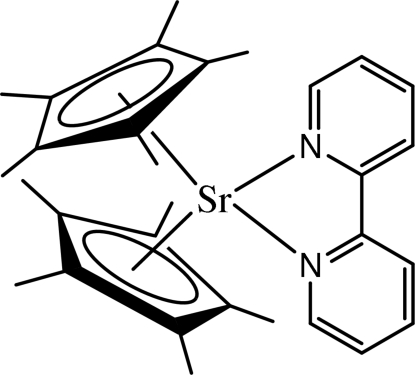


## Experimental

### 

#### Crystal data

[Sr(C_10_H_15_)_2_(C_10_H_8_N_2_)]
*M* *_r_* = 514.26
Orthorhombic, 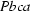

*a* = 15.5489 (9) Å
*b* = 16.7821 (9) Å
*c* = 20.561 (1) Å
*V* = 5365.4 (5) Å^3^
*Z* = 8
Mo *K*α radiation
μ = 2.03 mm^−1^
*T* = 168.2 K
0.10 × 0.09 × 0.03 mm

#### Data collection

Bruker APEX CCD diffractometer
Absorption correction: multi-scan (Blessing, 1995 ▶) *T*_min_ = 0.772, *T*_max_ = 0.941
30804 measured reflections
5478 independent reflections
3494 reflections with *F* ^2^ > 3σ(*F* ^2^)
*R*_int_ = 0.034

#### Refinement

*R*[*F* ^2^ > 2σ(*F* ^2^)] = 0.034
*wR*(*F* ^2^) = 0.038
*S* = 1.57
3494 reflections
298 parameters
H-atom parameters constrained
Δρ_max_ = 0.53 e Å^−3^
Δρ_min_ = −0.30 e Å^−3^

### 

Data collection: *SMART* (Bruker, 1999 ▶); cell refinement: *SAINT* (Bruker, 2002 ▶); data reduction: *SAINT*; program(s) used to solve structure: *SIR97* (Altomare *et al.*, 1999 ▶); program(s) used to refine structure: *TEXSAN* (MSC/Rigaku, 1998 ▶); molecular graphics: *TEXSAN*; software used to prepare material for publication: *TEXSAN*.

## Supplementary Material

Crystal structure: contains datablocks global, I. DOI: 10.1107/S1600536808024677/ww2113sup1.cif

Structure factors: contains datablocks I. DOI: 10.1107/S1600536808024677/ww2113Isup2.hkl

Additional supplementary materials:  crystallographic information; 3D view; checkCIF report

## Figures and Tables

**Table:** 

Sr1—N1	2.624 (3)
Sr1—N2	2.676 (3)
Sr1—*Cg*1	2.5711 (3)
Sr1—*Cg*2	2.5608 (3)

**Table:** 

C4⋯C27^i^	3.540 (5)
C9⋯C22^ii^	3.538 (5)
C12⋯C24^i^	3.589 (5)

**Table:** 

N1—C25—C26—N2	−2.2 (4)

Symmetry codes: (i) 
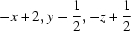
; (ii) 
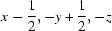
. *Cg*1 and *Cg*2 are the centroids of the C1–C5 and C6–C10 rings, respectively.

**Fig. 1.**
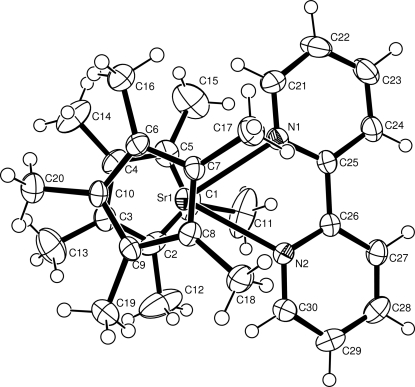
View of the structure of (η5-C5Me5)2Sr(bipy), showing the staggering of the two Cp* rings, with displacement ellipsoids drawn at the 50% probability level.

**Fig. 2.**
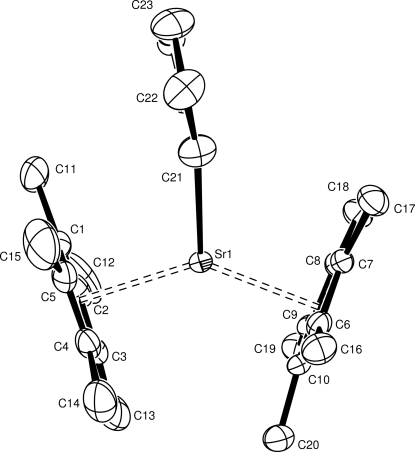
Side view of the structure of (η5-C5Me5)2Sr(bipy), with displacement ellipsoids drawn at the 50% probability level. Hydrogen atoms have been omitted for clarity.

## supplementary crystallographic information

### Comment

In Cp*_2_Sr(bipy) the Cp* rings are completely staggered (see Fig. 1). *Cg*1 and *Cg*2 are the centroids of the rings defined by C1—C5 and C6—C10 respectively. The two pyridine rings in the bipyridine ligand are almost coplanar (the N—C—C—N torsion angle is 2.2 (4)°). The least squares plane formed by the 12 atoms of the pyridine ring is tilted 10.7° relative to the plane formed by Sr,N1, and N2. This is larger than the equivalent angle in any other Cp*_2_M(bipy) in the Cambridge Structural Database (Allen, 2002). The closest is 5.1° in [Cp*_2_Yb(bipy)][Cp*_2_YbCl_2_] (Schultz *et al.* 2002). The reason for this tilting is unclear as the Sr is d^0^ and therefore electronic effects should be minor. At the same time it is unclear what steric reason could lead to this tilt.

### Experimental

Cp*_2_Sr(bipy) was prepared according to literature procedures (Burns and Andersen, 1987)

### Refinement

All non-hydrogen atoms were refined anisotropically. Hydrogen atoms were fixed based on the expected geometry of the carbon atoms to which they were attached.

### Crystal data

**Table:**

[Sr(C_10_H_15_)_2_(C_10_H_8_N_2_)]	*F*_000_ = 2160.00
*M**_r_* = 514.26	*D*_x_ = 1.273 Mg m^−^^3^
Orthorhombic, *P**b**c**a*	Mo *K*α radiation λ = 0.7107 Å
Hall symbol: -P 2ac 2ab	Cell parameters from 5093 reflections
*a* = 15.5489 (9) Å	θ = 2.4–25.2º
*b* = 16.7821 (9) Å	µ = 2.03 mm^−^^1^
*c* = 20.561 (1) Å	*T* = 168.2 K
*V* = 5365.4 (5) Å^3^	Plate, red
*Z* = 8	0.10 × 0.09 × 0.03 mm

### Data collection

**Table:**

Bruker APEX CCD diffractometer	3494 reflections with *F*^2^ > 3σ(*F*^2^)
ω scans	*R*_int_ = 0.034
Absorption correction: multi-scan(Blessing, 1995)	θ_max_ = 26.4º
*T*_min_ = 0.772, *T*_max_ = 0.941	*h* = 0→19
30804 measured reflections	*k* = 0→20
5478 independent reflections	*l* = 0→25

### Refinement

**Table:**

Refinement on *F*	H-atom parameters constrained
*R*[*F*^2^ > 2σ(*F*^2^)] = 0.034	*w* = 1/[σ^2^(*F*_o_) + 0.00022|*F*_o_|^2^]
*wR*(*F*^2^) = 0.039	(Δ/σ)_max_ = 0.002
*S* = 1.57	Δρ_max_ = 0.53 e Å^−^^3^
3494 reflections	Δρ_min_ = −0.30 e Å^−^^3^
298 parameters	Extinction correction: none

### Special details

**Table:**

Refinement. Refinement using reflections with *F*^2^ > 3.0 σ(*F*^2^). The weighted *R*-factor (*wR*), goodness of fit (*S*) and *R*-factor (gt) are based on *F*, with *F* set to zero for negative *F*. The threshold expression of *F*^2^ > 3.0 σ(*F*^2^) is used only for calculating *R*-factor (gt).

### Fractional atomic coordinates and isotropic or equivalent isotropic displacement parameters (Å^2^)

**Table:**

	*x*	*y*	*z*	*U*_iso_*/*U*_eq_	
Sr1	0.88103 (2)	0.21813 (2)	0.12421 (1)	0.02281 (7)	
N1	0.9677 (2)	0.3503 (2)	0.1051 (1)	0.0311 (8)	
N2	0.8425 (2)	0.3460 (1)	0.1963 (1)	0.0267 (8)	
C1	1.0006 (3)	0.1935 (2)	0.2267 (2)	0.041 (1)	
C2	0.9339 (2)	0.1399 (2)	0.2383 (2)	0.040 (1)	
C3	0.9370 (2)	0.0800 (2)	0.1897 (2)	0.034 (1)	
C4	1.0066 (2)	0.0990 (2)	0.1483 (2)	0.034 (1)	
C5	1.0460 (2)	0.1682 (2)	0.1715 (2)	0.038 (1)	
C6	0.8290 (2)	0.1915 (2)	−0.0059 (2)	0.0266 (9)	
C7	0.7927 (2)	0.2655 (2)	0.0097 (2)	0.0239 (9)	
C8	0.7266 (2)	0.2531 (2)	0.0561 (2)	0.0255 (9)	
C9	0.7230 (2)	0.1706 (2)	0.0694 (2)	0.0266 (9)	
C10	0.7861 (2)	0.1323 (2)	0.0310 (2)	0.0254 (9)	
C11	1.0229 (4)	0.2649 (3)	0.2689 (2)	0.084 (2)	
C12	0.8676 (4)	0.1433 (3)	0.2921 (2)	0.087 (2)	
C13	0.8814 (3)	0.0067 (3)	0.1861 (2)	0.063 (1)	
C14	1.0366 (3)	0.0512 (3)	0.0904 (2)	0.060 (1)	
C15	1.1271 (3)	0.2059 (3)	0.1456 (3)	0.075 (2)	
C16	0.8986 (2)	0.1763 (2)	−0.0554 (2)	0.039 (1)	
C17	0.8158 (2)	0.3448 (2)	−0.0202 (2)	0.039 (1)	
C18	0.6675 (2)	0.3164 (2)	0.0822 (2)	0.038 (1)	
C19	0.6609 (2)	0.1307 (2)	0.1157 (2)	0.039 (1)	
C20	0.7986 (2)	0.0439 (2)	0.0234 (2)	0.038 (1)	
C21	1.0290 (2)	0.3505 (2)	0.0594 (2)	0.039 (1)	
C22	1.0872 (3)	0.4116 (3)	0.0512 (2)	0.046 (1)	
C23	1.0803 (3)	0.4764 (2)	0.0915 (2)	0.048 (1)	
C24	1.0177 (2)	0.4781 (2)	0.1389 (2)	0.037 (1)	
C25	0.9619 (2)	0.4137 (2)	0.1450 (2)	0.0266 (9)	
C26	0.8938 (2)	0.4108 (2)	0.1962 (2)	0.0252 (9)	
C27	0.8846 (2)	0.4709 (2)	0.2424 (2)	0.0328 (10)	
C28	0.8216 (2)	0.4643 (2)	0.2889 (2)	0.041 (1)	
C29	0.7689 (2)	0.3982 (2)	0.2896 (2)	0.040 (1)	
C30	0.7819 (2)	0.3412 (2)	0.2421 (2)	0.035 (1)	
H1	0.9904	0.2628	0.3080	0.1013*	
H2	1.0825	0.2640	0.2789	0.1013*	
H3	1.0096	0.3126	0.2461	0.1013*	
H4	0.8535	0.0907	0.3054	0.1044*	
H5	0.8903	0.1720	0.3280	0.1044*	
H6	0.8173	0.1692	0.2766	0.1044*	
H7	0.8961	−0.0285	0.2205	0.0757*	
H8	0.8227	0.0215	0.1899	0.0757*	
H9	0.8904	−0.0193	0.1456	0.0757*	
H10	1.0396	−0.0035	0.1019	0.0724*	
H11	0.9971	0.0578	0.0556	0.0724*	
H12	1.0919	0.0692	0.0773	0.0724*	
H13	1.1757	0.1814	0.1653	0.0898*	
H14	1.1270	0.2612	0.1555	0.0898*	
H15	1.1297	0.1988	0.0998	0.0898*	
H16	0.9326	0.1322	−0.0419	0.0467*	
H17	0.8732	0.1649	−0.0963	0.0467*	
H18	0.9340	0.2222	−0.0591	0.0467*	
H19	0.8724	0.3422	−0.0375	0.0463*	
H20	0.7764	0.3569	−0.0541	0.0463*	
H21	0.8132	0.3852	0.0121	0.0463*	
H22	0.6148	0.2926	0.0954	0.0451*	
H23	0.6937	0.3418	0.1184	0.0451*	
H24	0.6565	0.3546	0.0492	0.0451*	
H25	0.6716	0.1488	0.1587	0.0473*	
H26	0.6036	0.1436	0.1036	0.0473*	
H27	0.6686	0.0746	0.1138	0.0473*	
H28	0.7707	0.0170	0.0582	0.0462*	
H29	0.8583	0.0319	0.0241	0.0462*	
H30	0.7746	0.0270	−0.0168	0.0462*	
H31	1.0326	0.3060	0.0310	0.0469*	
H32	1.1306	0.4089	0.0188	0.0555*	
H33	1.1186	0.5201	0.0867	0.0576*	
H34	1.0127	0.5227	0.1671	0.0441*	
H35	0.9215	0.5160	0.2417	0.0393*	
H36	0.8143	0.5051	0.3205	0.0490*	
H37	0.7252	0.3920	0.3215	0.0481*	
H38	0.7454	0.2958	0.2420	0.0414*	

### Atomic displacement parameters (Å^2^)

**Table:**

	*U*^11^	*U*^22^	*U*^33^	*U*^12^	*U*^13^	*U*^23^
Sr1	0.0253 (1)	0.0195 (1)	0.0236 (1)	−0.0008 (1)	−0.0028 (1)	−0.0020 (1)
N1	0.035 (2)	0.029 (2)	0.029 (2)	−0.006 (1)	0.003 (1)	−0.002 (1)
N2	0.027 (1)	0.025 (1)	0.028 (2)	0.002 (1)	0.001 (1)	−0.001 (1)
C1	0.053 (3)	0.032 (2)	0.037 (2)	0.013 (2)	−0.022 (2)	−0.007 (2)
C2	0.053 (3)	0.044 (2)	0.023 (2)	0.024 (2)	0.005 (2)	0.007 (2)
C3	0.036 (2)	0.027 (2)	0.038 (2)	0.005 (2)	−0.007 (2)	0.006 (2)
C4	0.037 (2)	0.036 (2)	0.028 (2)	0.016 (2)	−0.003 (2)	−0.002 (2)
C5	0.027 (2)	0.038 (2)	0.048 (2)	0.004 (2)	−0.010 (2)	0.004 (2)
C6	0.029 (2)	0.030 (2)	0.021 (2)	0.000 (1)	−0.002 (1)	−0.004 (1)
C7	0.030 (2)	0.021 (2)	0.021 (2)	−0.001 (1)	−0.003 (1)	0.002 (1)
C8	0.023 (2)	0.026 (2)	0.028 (2)	0.004 (1)	−0.004 (2)	−0.001 (1)
C9	0.027 (2)	0.024 (2)	0.029 (2)	−0.006 (1)	−0.002 (1)	0.000 (1)
C10	0.027 (2)	0.020 (2)	0.029 (2)	−0.001 (1)	−0.007 (1)	−0.005 (1)
C11	0.115 (5)	0.061 (3)	0.077 (4)	0.033 (3)	−0.066 (3)	−0.032 (3)
C12	0.115 (4)	0.097 (4)	0.049 (3)	0.066 (3)	0.038 (3)	0.037 (3)
C13	0.064 (3)	0.043 (3)	0.082 (3)	−0.005 (2)	−0.012 (3)	0.024 (2)
C14	0.063 (3)	0.074 (3)	0.044 (3)	0.035 (2)	−0.007 (2)	−0.015 (2)
C15	0.030 (2)	0.081 (4)	0.114 (4)	0.002 (2)	−0.011 (2)	0.024 (3)
C16	0.043 (3)	0.040 (2)	0.034 (2)	−0.001 (2)	0.003 (2)	−0.006 (2)
C17	0.048 (2)	0.032 (2)	0.036 (2)	0.002 (2)	0.000 (2)	0.006 (2)
C18	0.036 (2)	0.034 (2)	0.043 (2)	0.008 (2)	0.003 (2)	0.000 (2)
C19	0.037 (2)	0.037 (2)	0.044 (2)	−0.008 (2)	0.005 (2)	0.004 (2)
C20	0.039 (2)	0.031 (2)	0.045 (2)	−0.002 (2)	−0.005 (2)	−0.006 (2)
C21	0.044 (2)	0.040 (2)	0.033 (2)	−0.004 (2)	0.009 (2)	−0.006 (2)
C22	0.046 (2)	0.060 (3)	0.033 (2)	−0.017 (2)	0.011 (2)	0.001 (2)
C23	0.053 (3)	0.041 (3)	0.050 (3)	−0.022 (2)	0.008 (2)	0.001 (2)
C24	0.043 (2)	0.028 (2)	0.040 (2)	−0.007 (2)	0.001 (2)	−0.002 (2)
C25	0.028 (2)	0.023 (2)	0.029 (2)	−0.001 (1)	−0.004 (1)	0.002 (1)
C26	0.026 (2)	0.024 (2)	0.025 (2)	0.001 (1)	−0.006 (1)	0.002 (1)
C27	0.034 (2)	0.029 (2)	0.036 (2)	−0.002 (2)	−0.006 (2)	−0.006 (1)
C28	0.041 (2)	0.046 (2)	0.035 (2)	0.009 (2)	−0.003 (2)	−0.013 (2)
C29	0.034 (2)	0.052 (2)	0.035 (2)	0.008 (2)	0.006 (2)	−0.004 (2)
C30	0.033 (2)	0.033 (2)	0.038 (2)	−0.001 (2)	0.005 (2)	0.002 (2)

### Geometric parameters (Å, °)

**Table:**

SR1—N1	2.624 (3)	C21—C22	1.378 (5)
SR1—N2	2.676 (3)	C22—C23	1.370 (5)
SR1—C1	2.841 (3)	C23—C24	1.378 (5)
SR1—C2	2.812 (3)	C24—C25	1.392 (4)
SR1—C3	2.819 (3)	C25—C26	1.495 (4)
SR1—C4	2.838 (3)	C26—C27	1.393 (4)
SR1—C5	2.869 (3)	C27—C28	1.374 (5)
SR1—C6	2.830 (3)	C28—C29	1.380 (5)
SR1—C7	2.840 (3)	C29—C30	1.382 (5)
SR1—C8	2.841 (3)	C11—H1	0.950
SR1—C9	2.818 (3)	C11—H2	0.950
SR1—C10	2.815 (3)	C11—H3	0.950
SR1—Cg1	2.5711 (3)	C12—H4	0.950
SR1—Cg2	2.5608 (3)	C12—H5	0.950
N1—C21	1.339 (4)	C12—H6	0.950
N1—C25	1.346 (4)	C13—H7	0.950
N2—C26	1.349 (4)	C13—H8	0.950
N2—C30	1.334 (4)	C13—H9	0.950
C1—C2	1.394 (5)	C14—H10	0.950
C1—C5	1.402 (5)	C14—H11	0.950
C1—C11	1.520 (5)	C14—H12	0.950
C1—Cg1	1.189 (4)	C15—H13	0.950
C2—C3	1.419 (5)	C15—H14	0.950
C2—C12	1.512 (5)	C15—H15	0.950
C2—Cg1	1.199 (4)	C16—H16	0.950
C3—C4	1.412 (5)	C16—H17	0.950
C3—C13	1.506 (5)	C16—H18	0.950
C3—Cg1	1.207 (4)	C17—H19	0.950
C4—C5	1.396 (5)	C17—H20	0.950
C4—C14	1.510 (5)	C17—H21	0.950
C4—Cg1	1.189 (3)	C18—H22	0.950
C5—C15	1.508 (6)	C18—H23	0.950
C5—Cg1	1.190 (4)	C18—H24	0.950
C6—C7	1.402 (4)	C19—H25	0.950
C6—C10	1.416 (4)	C19—H26	0.950
C6—C16	1.508 (5)	C19—H27	0.950
C6—Cg2	1.199 (3)	C20—H28	0.950
C7—C8	1.418 (4)	C20—H29	0.950
C7—C17	1.509 (5)	C20—H30	0.950
C7—Cg2	1.197 (3)	C21—H31	0.950
C8—C9	1.412 (4)	C22—H32	0.950
C8—C18	1.503 (4)	C23—H33	0.950
C8—Cg2	1.206 (3)	C24—H34	0.950
C9—C10	1.413 (4)	C27—H35	0.950
C9—C19	1.513 (4)	C28—H36	0.950
C9—Cg2	1.203 (3)	C29—H37	0.950
C10—C20	1.505 (4)	C30—H38	0.950
C10—Cg2	1.201 (3)		
			
SR1···N1	2.624 (3)	SR1···C3	2.819 (3)
SR1···N2	2.676 (3)	SR1···C6	2.830 (3)
SR1···C1	2.841 (3)	SR1···C4	2.838 (3)
SR1···C2	2.812 (3)	SR1···C7	2.840 (3)
SR1···C3	2.819 (3)	SR1···C8	2.841 (3)
SR1···C4	2.838 (3)	SR1···C1	2.841 (3)
SR1···C5	2.869 (3)	SR1···C5	2.869 (3)
SR1···C6	2.830 (3)	SR1···C21	3.465 (4)
SR1···C7	2.840 (3)	SR1···C30	3.537 (4)
SR1···C8	2.841 (3)	SR1···C25	3.540 (3)
SR1···C9	2.818 (3)	SR1···C26	3.562 (3)
SR1···C10	2.815 (3)	N1···C17	3.497 (4)
SR1···Cg1	2.5711 (3)	N1···C5	3.562 (4)
SR1···Cg2	2.5608 (3)	N1···C15	3.565 (5)
SR1···Cg2	2.5608 (3)	N2···C11	3.455 (5)
SR1···Cg1	2.5711 (3)	C4···C27^i^	3.540 (5)
SR1···N1	2.624 (3)	C9···C22^ii^	3.538 (5)
SR1···N2	2.676 (3)	C11···C26	3.500 (5)
SR1···C2	2.812 (3)	C12···C24^i^	3.589 (5)
SR1···C10	2.815 (3)	C15···C21	3.371 (6)
SR1···C9	2.818 (3)		
			
N1—SR1—N2	61.32 (8)	SR1—C7—C17	118.0 (2)
N1—SR1—C1	84.1 (1)	SR1—C7—Cg2	64.4 (1)
N1—SR1—C2	111.7 (1)	C6—C7—C8	108.4 (3)
N1—SR1—C3	127.42 (9)	C6—C7—C17	126.3 (3)
N1—SR1—C4	105.55 (10)	C6—C7—Cg2	54.3 (2)
N1—SR1—C5	80.72 (9)	C8—C7—C17	125.2 (3)
N1—SR1—C6	97.99 (9)	C8—C7—Cg2	54.1 (2)
N1—SR1—C7	83.55 (9)	C17—C7—Cg2	177.6 (3)
N1—SR1—C8	100.70 (9)	SR1—C8—C7	75.5 (2)
N1—SR1—C9	128.80 (9)	SR1—C8—C9	74.7 (2)
N1—SR1—C10	126.83 (9)	SR1—C8—C18	119.2 (2)
N1—SR1—Cg1	102.33 (6)	SR1—C8—Cg2	64.3 (1)
N1—SR1—Cg2	108.44 (6)	C7—C8—C9	107.6 (3)
N2—SR1—C1	81.48 (9)	C7—C8—C18	125.4 (3)
N2—SR1—C2	88.69 (9)	C7—C8—Cg2	53.5 (2)
N2—SR1—C3	117.64 (9)	C9—C8—C18	126.9 (3)
N2—SR1—C4	128.44 (9)	C9—C8—Cg2	54.0 (2)
N2—SR1—C5	104.27 (9)	C18—C8—Cg2	176.3 (3)
N2—SR1—C6	125.91 (9)	SR1—C9—C8	76.4 (2)
N2—SR1—C7	97.29 (8)	SR1—C9—C10	75.4 (2)
N2—SR1—C8	85.32 (8)	SR1—C9—C19	115.5 (2)
N2—SR1—C9	104.69 (9)	SR1—C9—Cg2	65.3 (1)
N2—SR1—C10	132.08 (8)	C8—C9—C10	108.1 (3)
N2—SR1—Cg1	104.87 (6)	C8—C9—C19	125.5 (3)
N2—SR1—Cg2	110.07 (6)	C8—C9—Cg2	54.2 (2)
C1—SR1—C2	28.5 (1)	C10—C9—C19	126.4 (3)
C1—SR1—C3	47.4 (1)	C10—C9—Cg2	53.9 (2)
C1—SR1—C4	46.96 (10)	C19—C9—Cg2	179.2 (3)
C1—SR1—C5	28.4 (1)	SR1—C10—C6	76.0 (2)
C1—SR1—C6	149.9 (1)	SR1—C10—C9	75.6 (2)
C1—SR1—C7	166.5 (1)	SR1—C10—C20	120.5 (2)
C1—SR1—C8	161.6 (1)	SR1—C10—Cg2	65.4 (1)
C1—SR1—C9	145.7 (1)	C6—C10—C9	107.9 (3)
C1—SR1—C10	140.70 (10)	C6—C10—C20	125.2 (3)
C1—SR1—Cg1	24.75 (7)	C6—C10—Cg2	53.8 (2)
C1—SR1—Cg2	165.79 (7)	C9—C10—C20	126.5 (3)
C2—SR1—C3	29.20 (10)	C9—C10—Cg2	54.1 (2)
C2—SR1—C4	47.49 (10)	C20—C10—Cg2	174.0 (3)
C2—SR1—C5	47.1 (1)	N1—C21—C22	123.8 (3)
C2—SR1—C6	143.0 (1)	C21—C22—C23	117.7 (3)
C2—SR1—C7	164.6 (1)	C22—C23—C24	120.0 (3)
C2—SR1—C8	139.0 (1)	C23—C24—C25	119.2 (3)
C2—SR1—C9	117.2 (1)	N1—C25—C24	121.2 (3)
C2—SR1—C10	118.9 (1)	N1—C25—C26	116.8 (3)
C2—SR1—Cg1	25.23 (7)	C24—C25—C26	122.0 (3)
C2—SR1—Cg2	139.88 (9)	N2—C26—C25	116.5 (3)
C3—SR1—C4	28.92 (10)	N2—C26—C27	121.5 (3)
C3—SR1—C5	47.25 (10)	C25—C26—C27	122.0 (3)
C3—SR1—C6	114.19 (10)	C26—C27—C28	119.3 (3)
C3—SR1—C7	140.88 (10)	C27—C28—C29	119.7 (3)
C3—SR1—C8	131.80 (10)	C28—C29—C30	117.6 (3)
C3—SR1—C9	103.18 (9)	N2—C30—C29	124.1 (3)
C3—SR1—C10	93.82 (10)	SR1—Cg1—C1	90.4 (2)
C3—SR1—Cg1	25.35 (7)	SR1—Cg1—C2	88.7 (2)
C3—SR1—Cg2	118.38 (7)	SR1—Cg1—C3	88.9 (2)
C4—SR1—C5	28.31 (10)	SR1—Cg1—C4	90.3 (2)
C4—SR1—C6	104.51 (10)	SR1—Cg1—C5	91.9 (2)
C4—SR1—C7	132.45 (9)	C1—Cg1—C2	71.4 (3)
C4—SR1—C8	144.42 (10)	C1—Cg1—C3	143.7 (3)
C4—SR1—C9	118.07 (10)	C1—Cg1—C4	144.1 (3)
C4—SR1—C10	96.86 (9)	C1—Cg1—C5	72.2 (3)
C4—SR1—Cg1	24.78 (7)	C2—Cg1—C3	72.3 (3)
C4—SR1—Cg2	120.99 (7)	C2—Cg1—C4	144.5 (3)
C5—SR1—C6	122.0 (1)	C2—Cg1—C5	143.6 (3)
C5—SR1—C7	142.7 (1)	C3—Cg1—C4	72.2 (2)
C5—SR1—C8	169.5 (1)	C3—Cg1—C5	144.1 (2)
C5—SR1—C9	146.35 (10)	C4—Cg1—C5	71.8 (2)
C5—SR1—C10	123.37 (10)	SR1—Cg2—C6	90.1 (1)
C5—SR1—Cg1	24.49 (7)	SR1—Cg2—C7	90.7 (1)
C5—SR1—Cg2	144.63 (8)	SR1—Cg2—C8	90.5 (2)
C6—SR1—C7	28.63 (9)	SR1—Cg2—C9	89.4 (1)
C6—SR1—C8	47.57 (9)	SR1—Cg2—C10	89.3 (1)
C6—SR1—C9	47.77 (9)	C6—Cg2—C7	71.6 (2)
C6—SR1—C10	29.05 (9)	C6—Cg2—C8	144.0 (2)
C6—SR1—Cg1	128.95 (7)	C6—Cg2—C9	144.3 (2)
C6—SR1—Cg2	25.08 (6)	C6—Cg2—C10	72.3 (2)
C7—SR1—C8	28.92 (9)	C7—Cg2—C8	72.4 (2)
C7—SR1—C9	47.60 (9)	C7—Cg2—C9	144.1 (2)
C7—SR1—C10	47.55 (9)	C7—Cg2—C10	143.9 (2)
C7—SR1—Cg1	157.22 (7)	C8—Cg2—C9	71.7 (2)
C7—SR1—Cg2	24.93 (6)	C8—Cg2—C10	143.8 (2)
C8—SR1—C9	28.89 (9)	C9—Cg2—C10	72.0 (2)
C8—SR1—C10	47.71 (9)	C1—C11—H1	109.470
C8—SR1—Cg1	156.95 (7)	C1—C11—H2	109.470
C8—SR1—Cg2	25.12 (7)	C1—C11—H3	109.470
C9—SR1—C10	29.06 (9)	H1—C11—H2	109.472
C9—SR1—Cg1	128.52 (6)	H1—C11—H3	109.472
C9—SR1—Cg2	25.27 (6)	H2—C11—H3	109.473
C10—SR1—Cg1	116.13 (6)	C2—C12—H4	109.470
C10—SR1—Cg2	25.25 (6)	C2—C12—H5	109.470
Cg1—SR1—Cg2	141.38 (1)	C2—C12—H6	109.470
SR1—N1—C21	118.3 (2)	H4—C12—H5	109.472
SR1—N1—C25	122.9 (2)	H4—C12—H6	109.472
C21—N1—C25	118.2 (3)	H5—C12—H6	109.473
SR1—N2—C26	120.9 (2)	C3—C13—H7	109.470
SR1—N2—C30	120.1 (2)	C3—C13—H8	109.470
C26—N2—C30	117.8 (3)	C3—C13—H9	109.471
SR1—C1—C2	74.6 (2)	H7—C13—H8	109.472
SR1—C1—C5	76.9 (2)	H7—C13—H9	109.473
SR1—C1—C11	117.2 (2)	H8—C13—H9	109.472
SR1—C1—Cg1	64.8 (1)	C4—C14—H10	109.469
C2—C1—C5	108.5 (3)	C4—C14—H11	109.470
C2—C1—C11	125.5 (4)	C4—C14—H12	109.470
C2—C1—Cg1	54.6 (2)	H10—C14—H11	109.474
C5—C1—C11	125.9 (4)	H10—C14—H12	109.471
C5—C1—Cg1	53.9 (2)	H11—C14—H12	109.473
C11—C1—Cg1	177.9 (3)	C5—C15—H13	109.470
SR1—C2—C1	76.9 (2)	C5—C15—H14	109.470
SR1—C2—C3	75.7 (2)	C5—C15—H15	109.469
SR1—C2—C12	113.1 (2)	H13—C15—H14	109.473
SR1—C2—Cg1	66.1 (1)	H13—C15—H15	109.473
C1—C2—C3	108.1 (3)	H14—C15—H15	109.473
C1—C2—C12	127.5 (4)	C6—C16—H16	109.470
C1—C2—Cg1	54.0 (2)	C6—C16—H17	109.471
C3—C2—C12	124.4 (4)	C6—C16—H18	109.470
C3—C2—Cg1	54.1 (2)	H16—C16—H17	109.471
C12—C2—Cg1	178.3 (4)	H16—C16—H18	109.472
SR1—C3—C2	75.1 (2)	H17—C16—H18	109.473
SR1—C3—C4	76.3 (2)	C7—C17—H19	109.470
SR1—C3—C13	118.1 (2)	C7—C17—H20	109.470
SR1—C3—Cg1	65.8 (1)	C7—C17—H21	109.470
C2—C3—C4	106.9 (3)	H19—C17—H20	109.472
C2—C3—C13	126.4 (4)	H19—C17—H21	109.473
C2—C3—Cg1	53.6 (2)	H20—C17—H21	109.471
C4—C3—C13	126.5 (4)	C8—C18—H22	109.470
C4—C3—Cg1	53.3 (2)	C8—C18—H23	109.469
C13—C3—Cg1	176.1 (3)	C8—C18—H24	109.470
SR1—C4—C3	74.8 (2)	H22—C18—H23	109.472
SR1—C4—C5	77.1 (2)	H22—C18—H24	109.473
SR1—C4—C14	116.7 (2)	H23—C18—H24	109.473
SR1—C4—Cg1	65.0 (1)	C9—C19—H25	109.471
C3—C4—C5	108.6 (3)	C9—C19—H26	109.470
C3—C4—C14	126.2 (4)	C9—C19—H27	109.471
C3—C4—Cg1	54.5 (2)	H25—C19—H26	109.472
C5—C4—C14	125.2 (4)	H25—C19—H27	109.472
C5—C4—Cg1	54.1 (2)	H26—C19—H27	109.472
C14—C4—Cg1	178.2 (3)	C10—C20—H28	109.470
SR1—C5—C1	74.7 (2)	C10—C20—H29	109.469
SR1—C5—C4	74.6 (2)	C10—C20—H30	109.470
SR1—C5—C15	120.4 (3)	H28—C20—H29	109.473
SR1—C5—Cg1	63.6 (1)	H28—C20—H30	109.473
C1—C5—C4	107.9 (3)	H29—C20—H30	109.473
C1—C5—C15	125.4 (4)	N1—C21—H31	118.110
C1—C5—Cg1	53.9 (2)	C22—C21—H31	118.110
C4—C5—C15	126.5 (4)	C21—C22—H32	121.146
C4—C5—Cg1	54.1 (2)	C23—C22—H32	121.146
C15—C5—Cg1	176.0 (3)	C22—C23—H33	120.023
SR1—C6—C7	76.1 (2)	C24—C23—H33	120.023
SR1—C6—C10	74.9 (2)	C23—C24—H34	120.398
SR1—C6—C16	117.4 (2)	C25—C24—H34	120.399
SR1—C6—Cg2	64.8 (1)	C26—C27—H35	120.349
C7—C6—C10	108.0 (3)	C28—C27—H35	120.348
C7—C6—C16	126.4 (3)	C27—C28—H36	120.163
C7—C6—Cg2	54.1 (2)	C29—C28—H36	120.164
C10—C6—C16	125.6 (3)	C28—C29—H37	121.225
C10—C6—Cg2	53.9 (2)	C30—C29—H37	121.224
C16—C6—Cg2	177.7 (3)	N2—C30—H38	117.935
SR1—C7—C6	75.3 (2)	C29—C30—H38	117.936
SR1—C7—C8	75.6 (2)		

Symmetry codes: (i) −*x*+2, *y*−1/2, −*z*+1/2; (ii) *x*−1/2, −*y*+1/2, −*z*.
